# Fragmentary Blue: Resolving the Rarity Paradox in Flower Colors

**DOI:** 10.3389/fpls.2020.618203

**Published:** 2021-01-15

**Authors:** Adrian G. Dyer, Anke Jentsch, Martin Burd, Jair E. Garcia, Justyna Giejsztowt, Maria G. G. Camargo, Even Tjørve, Kathleen M. C. Tjørve, Peter White, Mani Shrestha

**Affiliations:** ^1^School of Media and Communication, RMIT University, Melbourne, VIC, Australia; ^2^Department of Disturbance Ecology, Bayreuth Center for Ecology and Environmental Research, University of Bayreuth, Bayreuth, Germany; ^3^School of Biological Sciences, Monash University, Melbourne, VIC, Australia; ^4^Phenology Lab, Biosciences Institute, Department of Biodiversity, UNESP – São Paulo State University, São Paulo, Brazil; ^5^Inland Norway University of Applied Sciences, Lillehammer, Norway; ^6^Department of Biology, The University of North Carolina at Chapel Hill, Chapel Hill, NC, United States; ^7^Faculty of Information Technology, Monash University, Melbourne, VIC, Australia

**Keywords:** blue, flower color, biogeography, elevation, land use, plant diversity, productivity

## Abstract

Blue is a favored color of many humans. While blue skies and oceans are a common visual experience, this color is less frequently observed in flowers. We first review how blue has been important in human culture, and thus how our perception of blue has likely influenced the way of scientifically evaluating signals produced in nature, including approaches as disparate as Goethe’s Farbenlehre, Linneaus’ plant taxonomy, and current studies of plant-pollinator networks. We discuss the fact that most animals, however, have different vision to humans; for example, bee pollinators have trichromatic vision based on UV-, Blue-, and Green-sensitive photoreceptors with innate preferences for predominantly short-wavelength reflecting colors, including what we perceive as blue. The subsequent evolution of blue flowers may be driven by increased competition for pollinators, both because of a harsher environment (as at high altitude) or from high diversity and density of flowering plants (as in nutrient-rich meadows). The adaptive value of blue flowers should also be reinforced by nutrient richness or other factors, abiotic and biotic, that may reduce extra costs of blue-pigments synthesis. We thus provide new perspectives emphasizing that, while humans view blue as a less frequently evolved color in nature, to understand signaling, it is essential to employ models of biologically relevant observers. By doing so, we conclude that short wavelength reflecting blue flowers are indeed frequent in nature when considering the color vision and preferences of bees.

## Introduction

Why make so much of fragmentary blue

In here and there a bird, or butterfly,

Or flower, or wearing-stone, or open eye,

When heaven presents in sheets the solid hue?

Since earth is earth, perhaps, not heaven (as yet)–

Though some savants make earth include the sky;

And blue so far above us comes so high,

*It only gives our wish for blue a whet.* (Frost, 1920)

When we as human observers use our color vision to document the natural world, we need to be cognizant of the limitations and biases of our perception. In this synthesis review, we consider the reported relative rarity of blue flowers in many ecological studies, and subsequently discuss how a different view of flower spectral data can be obtained by considering the vision of major pollinators with the goal of bridging different fields to navigate toward the frontiers of plant color science.

Studies in psychophysics on adult humans from many countries show that blue is the most frequently preferred color hue (Granger, 1952; McManus et al., 1981; Ou et al., 2004). A blue color preference is also observed in human infant studies (Teller et al., 2004; Zemach et al., 2007), although infant color vision experiments show evidence for a preference to reddish hues in some contexts (Franklin et al., 2010). An ecological explanation for our blue preference is that we like clear sky and blue water and increasingly develop a preference for those from young childhood (Palmer and Schloss, 2010), and indeed color preferences in humans are frequently influenced by important environmental factors in our lives (Palmer et al., 2013).

The color blue has long been highly valued throughout the history of humans. In ancient Egypt, the combination of silica (SiO_2_), calcium oxide (CaO), and copper oxide (CuO) was used to make Egyptian blue (*irtyu*), a long-lasting entrancing pigment representing the color of the sky and heavens that was used for the decoration of statues that can still be observed (Eastaugh et al., 2004). The earliest known use of blue dyes can be traced to ancient Peru where an indigoid dye (indigotin E132), was used to dye cotton fabric about 6000 years ago, about 1500 years before the first evidences of usage of blue fabric dyes in ancient Egypt (Splitstoser et al., 2016). Indigo blue dyes have been important in driving economics through the production of dyer’s weed, *Isatis tinctoria* and its economic rival, *Indigofera tinctoria*, which emerged with the expansion of European trade routes to India (Asiaticus, 1912; Sandberg, 1989; Clark et al., 1993). The presence of blue in nature inspired artists such as Michaelangelo, Albrecht Duerer, Gauguin, Picasso, and van Gough who used blue pigments like lapis lazuli or organic pigments. Blue is also used to represent important religious symbols such as the Hindu deity Lord Krishna (Blurton, 1993; Prabhupada Bhaktivedanta Swami, 2013, Bhagat Gita English edition) and the Virgin Mary (Pastoureau, 2001; Heller, 2008; Fallon, 2014). The relative scarcity of blue available in natural pigments likely fueled our fascination with the preferred color of many humans.

Color perception in humans is enabled by our trichromatic visual system containing photoreceptors maximally absorbing radiation of wavelengths about 421, 530, and 559 nm (blue, green, and red). Our visual system compares the responses of these photoreceptors by means of an opponent system (Hurvich, 1981; Koenderink, 2010), which can also be influenced by a variety of ecological and contextual effects (Palmer et al., 2013). A more detailed explanation of comparative color vision is provided in the second part of this work, but first we address how color as a trait has typically been employed for classifying plants as a baseline to our current understanding. To better understand how we or other animals use spectral information requires care and consideration of the context of how color may work differently depending upon the observers and their visual experience (Palmer et al., 2013; Kemp et al., 2015).

**FIGURE 1:**
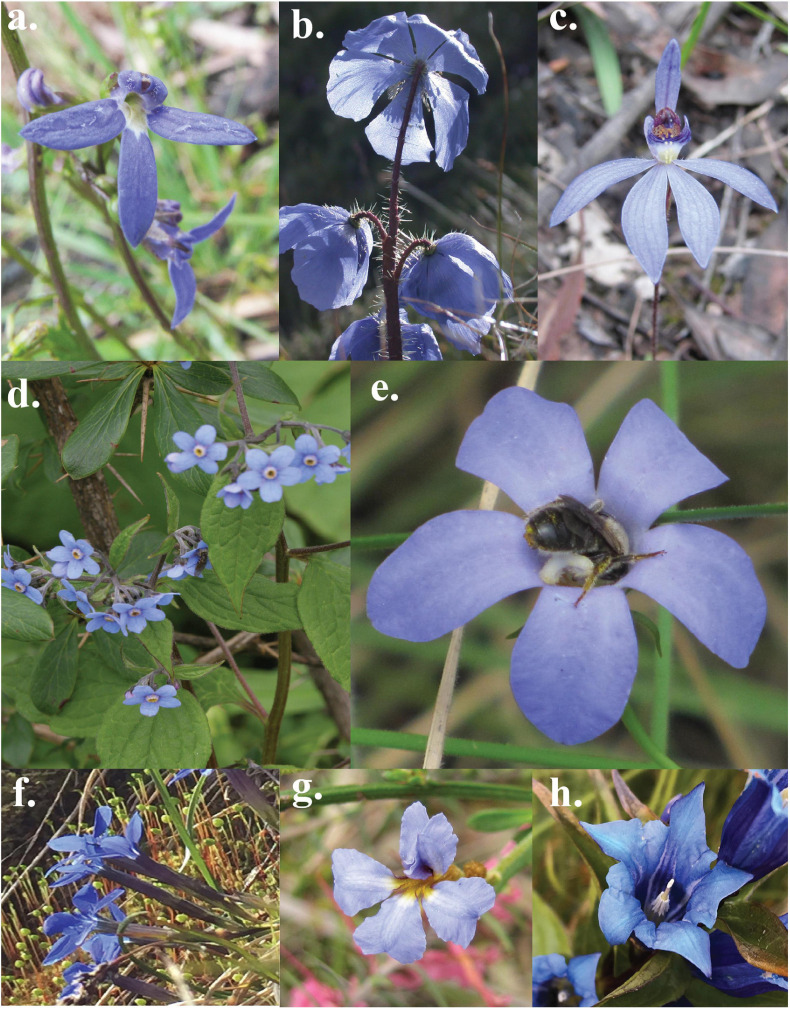
Example of some flowers perceived as blue by a human observer: **(a)** *Lobelia rhombifolia*, **(b)** *Meconopsis horridula*, **(c)** *Cyanicula caerulea*, **(d)** *Hackelia uncinata*, **(e)** *Wahlenbergia gloriosa*, **(f)** *Gentiana bavarica*, **(g)** *Dampiera stricta*, and **(h)** *Gentiana asclepiadea* (Image Credit: Anke Jentsch, Mani Shrestha).

**FIGURE 2:**
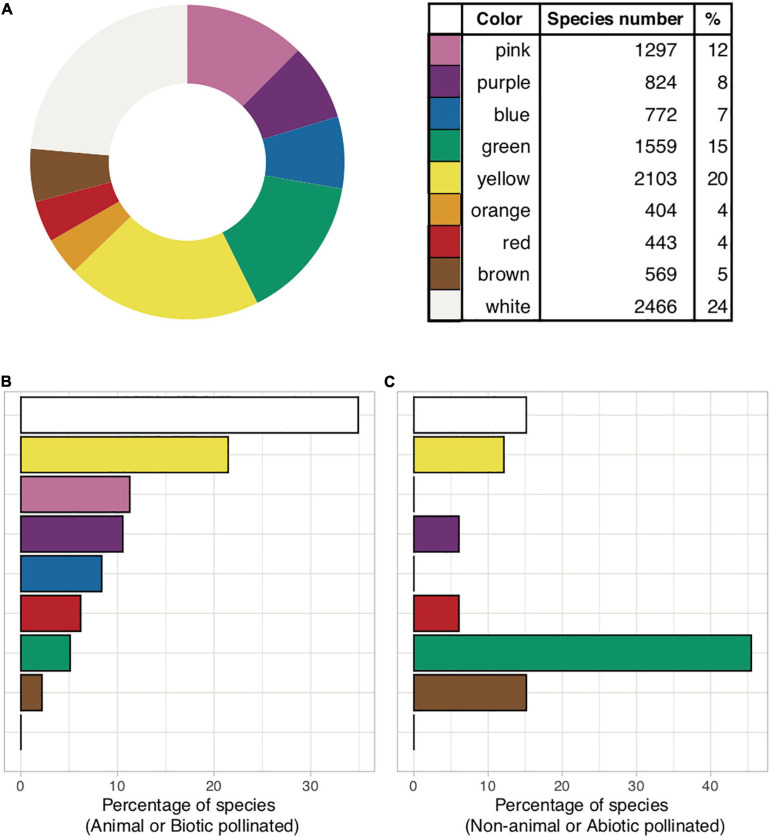
**(A)** Global flower color frequency based on human visual perception (*n* = 10,437 species; data source: Kattge et al., 2020, https://www.try-db.org/TryWeb/). **(B)** Flower color frequency in animal pollinated species (*n* = 275) where less than 10% are blue. **(C)** Non-animal pollinated species (*n* = 33), where blue does not occur. All wind-pollinated angiosperms are secondarily wind-pollinated and thus the color of these flowers might be relicts from a previous animal pollinated period. **(B,C)** are limited to European data only. R code available in Supplementary Appendix 1.

While much of human history is surrounded by blue skies and waters, this fascination may nevertheless have also emerged from its perceived rarity in the biological world (Goethe, 1810: “Farbenlehre”). A blue flower was a central symbol of inspiration for the Romanticism movement in Europe (Novalis, 1802) and remains important in contemporary Western art (Gage, 1999). In fact, flowers perceived by humans as being blue (Figure 1) are infrequent, constituting less than 10% of the nearly 300,000 known species of flowering plants (Lee, 2010). Blue flowers are also reported to be phylogenetically restricted, occurring in only 372 out of 14,038 genera of angiosperms worldwide, and in 53 out of 406 plant families (Gottsberger and Gottlieb, 1981). Considering available data in the newly extended international plant trait database “TRY database” (Kattge et al., 2020), an overall 772 of 10,437 (7%) species are classified as being “blue” flowers, with other human-perceived colors being more frequent (Figure 2 and Supplementary Figure 1). Interestingly, blue is far more common among biotically-pollinated flowers in the TRY database than among wind-pollinated flowers, where blue colors are almost non-existent, although in general wind pollinated flowers lack salient colors (Figure 2). These data suggest that exploring the rarity of blue flowers requires a deeper understanding of how animals perceive these colors. Flower color plays an important role in the taxonomy of plants by helping differentiate between single species (Linnaeus, 1735, 1755, 1785) as well as in the ecology of plants by attracting pollinators (Chittka and Menzel, 1992; Shrestha et al., 2013; Ohashi et al., 2015; LeCroy et al., 2021). In addition, other factors can also be at play, as recent evidence suggests that increased pollinator competition may also promote convergence toward the most preferred colors (Shrestha et al., 2019a; Tai et al., 2020), which is discussed in depth below. Accordingly, in harsher conditions, with less competition, higher divergence of flower colors is observed (Dalrymple et al., 2020).

Surprisingly, it is not only humans for which short wavelength blue coloration has a special allure: honeybees (Morawetz et al., 2013), bumblebees (Gumbert, 2000), and stingless bees (Dyer et al., 2016a) have innate preferences for short wavelength blue colors. This leads to the important question of why blue is not more frequently observed among flower colors in nature, and, indeed, whether this impression of blue scarcity is correct. This multidisciplinary research question draws attention to fields as diverse as the biochemistry of floral pigments, land-use management, and biodiversity research, as well as the biogeography of available resources in different environments, and how humans and pollinators perceive color. We discuss these factors (biochemistry of blue pigment evolution and pollinators, developmental and environmental controls of blue, nutrient availability and plant diversity with respect to blue flowering species, land use intensity as a driver of flower color richness, water availability, and drought stress) below to provide bridges into our comparative understanding of flower color evolution, and how blue flowers might be an important biomarker of complex factors influencing biodiversity.

## Biochemistry of Blue Pigment Evolution and Pollinators

Flower colors are mainly determined by the chemical structure of anthocyanins (ancient Greek for “blue flower”), a group of flavonoids (Grotewold, 2006; Katsumoto et al., 2007; Tanaka et al., 2008). Although the synthetic pathway for anthocyanin can yield several alternative forms (Lee, 2007), the majority of blue flowers contain delphinidin-based anthocyanins (Honda and Saito, 2002). The pH in the vacuole, where anthocyanins are localized, can also alter the color of anthocyanins, with blue colors produced in a weakly acidic or neutral cellular environment (Goto and Kondo, 1991). Stacking of co-occurring pigments like flavones or flavonols with anthocyanins or the formation of a complex with metal ions (Fe 3^+^, Mg 2^+^, 2 Ca2^+^) can be key elements for the production of blue flowers (Kondo et al., 1992; Yoshida et al., 2003; Shiono et al., 2005; Shoji et al., 2007). It takes a complicated chemical pathway to generate blue flower color, where six anthocyanins together with six co-occurring molecules form a ring around two central metal ions. With a multitude of potential mechanisms for modifying anthocyanin pigments to produce blue colors (Lee, 2007), it remains unclear what inhibits plant species from expressing blue flower coloration (Yoshida et al., 2009). This is especially true when blue coloration may also be achieved via structural coloration (Vignolini et al., 2015; Moyroud et al., 2017), and/or a mixture of pigment and structural colors (van der Kooi and Stavenga, 2019).

Anthocyanins comprise three major types: pelargonidin (generally red), cyanidin (typically magenta or blue depending on pH), and delphinidin (generally blue) (Mol et al., 1996; Davies, 2004). These pigments play an important role attracting fauna for pollination, seed dispersal, protection against stress, and signaling (reviewed by Koes et al., 2005). Research has shown that bird-pollinated flowers are much more likely to contain pelargonidin and much less likely to contain delphinidin than other flowers (Scogin, 1988; Davies, 2004), whereas insect-visited plants may contain flavonoid, delphinidin, cyanidin, and carotinoid pigmentation (Davies, 2004; Samanta et al., 2011). Plant pigments and pollinator groups are classified according to classical pollination syndromes as perceived by humans (Davies, 2004). Furthermore, Smith and Rausher (2011) reported shifting production of delphinidin to pelargonidin in *Ipomea gesnerioide*, which shows that the bird-pollinated flowers are most frequently evolved from bee-pollinated plants. Thus, floral color can evolve through quantitative variation in the production of several types of pigments (Davies, 2004; Kellenberger et al., 2019), leading to complex floral reflectance spectra (Davies, 2004; van der Kooi et al., 2016). Recent work also shows that the potential color effect of pigments can be further modified by the distribution of pigment cells in the structure of the flower petal due to optical effects (van der Kooi et al., 2016), suggesting that flowers potentially have a number of biologically plausible mechanisms to tune or modulate color signals.

## Developmental and Environmental Controls

Anthocyanin production can be induced by both developmental and environmental controls (Farzad et al., 2003). For instance, temperature or ultraviolet light intensity can influence the floral anthocyanin content (Mol et al., 1996; Lu et al., 2009) and therefore the intensity of flower colors. Additionally, resource restriction such as cold temperature, a lack of nitrogen (Do and Cormier, 1991; Rajendran et al., 1992; Bongue-Bartelsman and Phillips, 1995) or phosphorus, exposure to lower pH (Suzuki, 1995), stress such as wounding (Ferreres et al., 1997), or pathogen infection (Dixon et al., 1994) may increase anthocyanin production (Chalker-Scott, 1999). In addition, certain nutrients, in particular aluminum Al^3+^, combined with a low soil pH can induce a color change from purple to blue in some plant species (Chalker-Scott, 1999). Plant species can accumulate only limited kinds of anthocyanins and therefore there may be limits on the production of some flower colors by the expression of a specific set of biosynthetic genes (Katsumoto et al., 2007). As a result, in some plant families like roses, carnations and chrysanthemum no blue flowers occur naturally due to the lack of a key enzyme in the synthesis of delphinidin. In comparison, blue flowers occur in those taxa of angiosperms that have a higher proportion of herbaceous species, which are mainly insect pollinated. Blue flowers are thought to be rare in early diverging lineages, which are rather associated with wind pollination (Figure 2), and more frequent in derived groups like Asteridae, Commelinidae and some clades of Liliidae like *Linum* sections *Linum* and *Dasylinum* (McDill et al., 2009). Even with sophisticated genetic engineering techniques, it was extremely difficult to modify the colors of “white” roses to reflect blue wavelengths of light (Ogata et al., 2005; Tanaka et al., 2009), a challenge that would have considerable commercial benefits due to human aesthetics and color preferences, and perhaps requiring changes to popular poems and tunes using the verse “*Les bluets sont bleus, les roses sont roses*,” immortalized in Victor Hugo’s novel “Les Misérables” (Hugo, 1863, p. 97), and its English equivalent, “Roses are Red, Violets are Blue.”

## Nutrient Availability and Plant Diversity

**FIGURE 3:**
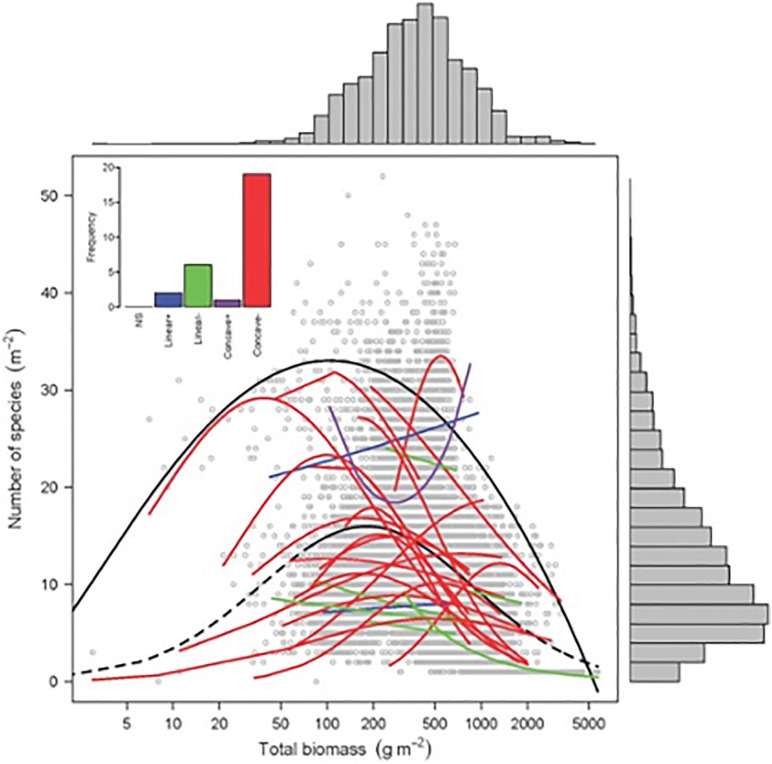
Biomass–richness relationship for 28 grasslands across the world. Black solid line: significant quantile regression (95%) of overall relationship (*P* < 0.001; *n* = 9631 quadrats). Colored lines: significant GLM regressions (Poisson or quasi-Poisson), with *N* ranging from 128 to 894 (Source: Fraser et al., 2015). Reprinted with permission from AAAS, permission no: 4976850603684.

Finding patterns that drive diversity in plant systems and in plant communities is a major issue in plant ecology. Emerging questions include how productivity affects species richness and trait distribution in plant communities. The humped-back model (HBM) (Grime, 1973) suggests that plant species richness peaks at intermediate productivity, taking above-ground biomass as a proxy for annual net primary productivity. This diversity peak is driven by two opposing processes (Figure 3, Fraser et al., 2015). In unproductive and disturbed ecosystems with low plant biomass, species richness is limited by either abiotic stress, such as insufficient water and mineral nutrients, or high levels of disturbance-induced biomass removal, which few species are able to tolerate. In contrast, in the low disturbance and productive conditions that generate high plant biomass, exclusion by a small number of highly competitive species is hypothesized to constrain species richness. Other mechanisms that may explain the unimodal relationship between species richness and productivity include disturbance (Connell, 1978; Jentsch and White, 2019), evolutionary history, and dispersal limitation (Taylor, 1990; Zobel and Pärtel, 2008).

**FIGURE 4:**
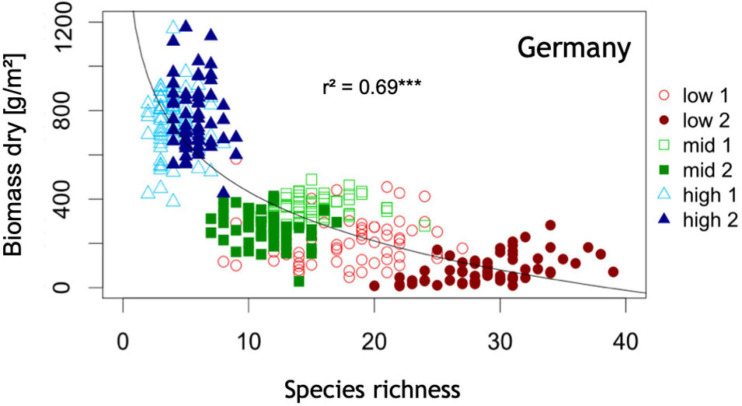
Plant species richness as a function above ground biomass production: Highest community plant species richness is associated with lowest community productivity at this half of the productivity gradient. Low = site of low productivity, mid = sites of intermediate productivity, high = sites of high productivity. Data shown here are based on 364 plots of 1 m × 1 m area organized in six systematic grids across a local productivity gradient in mesic temperate grassland close to Bayreuth University in Germany, part of the Herbaceous Diversity Network HERBDIVNET (see details in Fraser et al., 2015). ***p < 0.001.

Additional factors including nutrient availability (Figure 4) (Bedford et al., 1999; Fraser et al., 2015) and habitat disturbance (Grime, 1973; Jentsch and White, 2019) may also influence flower color and plant species diversity (Figure 4), although at present causal mechanisms remain largely unknown. With increasing species richness, the percentage of blue flowering species has been reported to increase (Ostler and Harper, 1978; Schemske and Bierzychudek, 2007). In mesotrophic grasslands, blue flowers are reported to be absent from the most species-poor communities (Warren and Billington, 2005). Generally, co-flowering species increase the flower color complementarity and diversification of a community (Lázaro et al., 2009; de Jager et al., 2011; Makino and Yokoyama, 2015; Losapio et al., 2017). It is thought to be potentially advantageous to stand out from flowers of competitive species (Makino and Yokoyama, 2015) and also from the background against which flowers are viewed (Bukovac et al., 2017b) in order to attract pollinators, although continental surveys (Chittka and Menzel, 1992; Dyer et al., 2012; Bukovac et al., 2017a) and community studies (Kantsa et al., 2017, 2018; Shrestha et al., 2019b) reveal that plant flowers frequently converge to preferred signals of particular pollinators. This suggests that either high plant diversity may drive increased flower color diversity as a means of attracting specialist pollinators (Weiner et al., 2011; Mesgaran et al., 2017), and/or that blue flower color is viable environmentally in places that promote or require high diversity.

## Land Use Intensity and Flower Color Diversity

**FIGURE 5:**
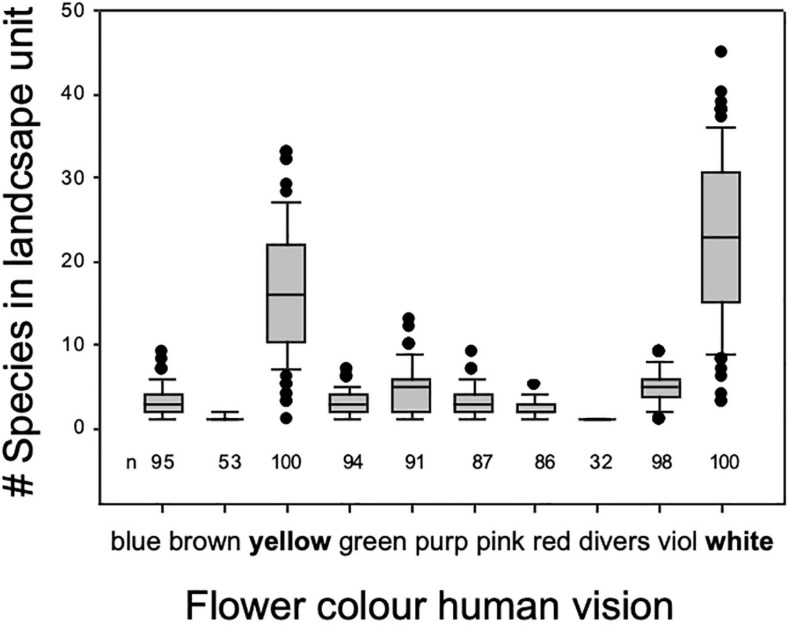
Number of plant species displaying flower color by 10 categories as sensed by human vision in a typical semi-natural, montaneous landscape in central Europe. Data are based on 100 plots of 100 m × 100 m systematically arranged with a 4 km × 4 km landscape unit a calcareous bedrock harboring mesic grasslands, small deciduous forests, hedges, rocky outcrops, partly grazed, partly mown, partly logged in the Franconian Swiss, Bavaria, Southern Germany regions. Most flowering forbs in this diver’s cultural landscape are perceived yellow or white by humans.

**FIGURE 6:**
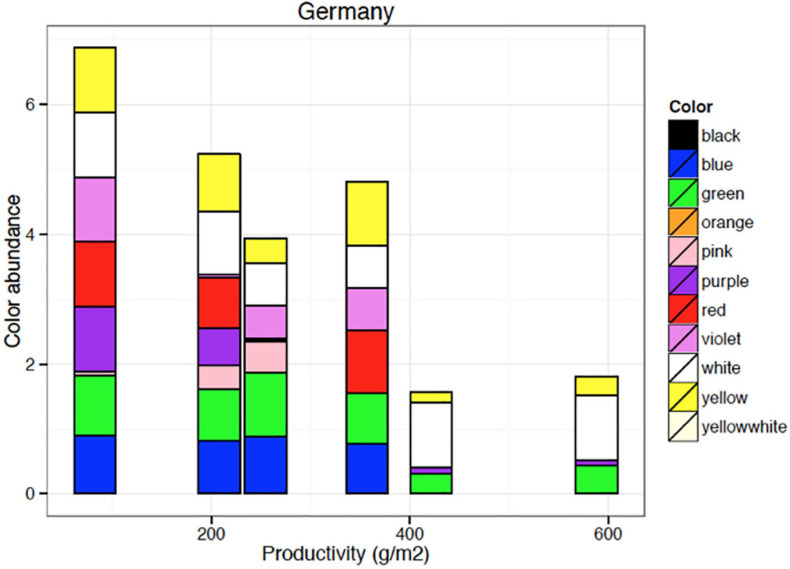
Flower color abundance of plant species growing on sites of various productivity. Blue and red flowering species (as seen by human vision) disappear from the plant communities with increasing site productivity. Data shown here are based on 364 plots of 1 m × 1 m area organized in six systematic grids across a local productivity gradient in mesic temperate grassland close to Bayreuth University in Germany, part of the Herbaceous Diversity Network HERBDIVNET (see details in Fraser et al., 2015).

Diversity is often driven by land-use intensity (Collins et al., 1998). Increasing land-use intensity results in fewer species as well as lower flower color species richness (Warren and Billington, 2005; Binkenstein et al., 2013) (Figures 5, 6). Increasing land-use intensity, measured in an index combining fertilization, grazing, and mowing, produced a shift in flower colors from less frequent blue toward mainly white flowering species as perceived by a human with normal color vision (Binkenstein et al., 2013). Specific effects of fertilization, grazing, or mowing could not be identified, and likely require more powerful models that incorporate the diversity of pollinator mediated influences.

In Scandinavia and the rest of Northern Europe all the way down to Bavaria in Southern Germany, the traditional, extensively cultivated flowering meadows are found in the lowlands and in the mountains, although in the lowland’s meadows increasing habitation is changing the environment in complex and unknown ways. Cultivated flower meadows seem to have an unusually high percentage of species with blue flowers (see, e.g., Stabbetorp and Endrestøl, 2011; Austad et al., 2015), and reduced meadows cause the demise of species possessing blue flowers such as *Dracocephalum ryschiana*, *Campanula barbata*, *Campanula rotundifolia*, *Jasione montana*, *Polygala serpyllifolia*, *Polygala amarella*, *Gentiana pneumonante*, *Gentiana nivalis* (and several other *Gentiana* species), *Hepatica nobilis* [*Anemone hepatica*], and *Viola hiarta* (as well as the more common violets). These meadows are threatened by climate warming, fertilization, and increased mowing frequency (Berauer et al., 2019) or from transition to modern farmland, development, or simply falling into fallow and eventually being taken over by forest. The decrease in small flowering species has also been associated with increase in nitrogen and phosphorus, which causes competition from large grasses and herbs (e.g., Stevens et al., 2004). These flowering species are typically found on unfertilized hayfields and pastures, especially on calcareous soils, which facilitates mineral uptake. In addition, such soils are often rich in minerals such as potassium, magnesium, and iron.

Some works suggest that many rare poorly competitive species with blue flowers may lose out to competition from larger plants. For example, blue-flowered species, such as violets (*Viola* sp.) (Jeffrey and Pigott, 1973; Maskell et al., 2010) and *C. rotundifolia* (Stevens et al., 2004; Maskell et al., 2010), are shown to be lost when the nitrogen and/or phosphorus content increases. Some small blue-flowered species may fail even from the actual increased phosphorus and/or nitrogen, as is reported for *J. montana* (Tyler, 1992). Also, the increased deposition of atmospheric nitrogen, which is quite low, may be a threat to small blue-flowered grassland species (see Stevens et al., 2004, 2011). Of other environmental changes, increased soil acidity from precipitation may also affect these low-growing calciphiles, as reported for *H. nobilis* (or *A. hepatica*) (Tyler et al., 2002).

Ekstam and Forshed (1992) provided a comprehensive list of nitrogen tolerance in plants and whether they are weak competitors. They note, for example, *Viola*, *Euphrasia*, *Polygala*, and some *Veronica* species to be particularly weak competitors with very low tolerance to nitrogen increase. Thus, many species with blue flowers show some evidence of being susceptible to land-use changes, especially since they are expected to be restricted to low nitrogen and low phosphorus but high micronutrient (calcium and other) environments. It is also worth asking whether the presence of blue flower may act as biomarkers of healthy land/ecosystem that have intense land use pattern with uses of different fertilizers.

## Water Availability and Drought Stress

Other factors such as water stress may influence the occurrence of blue flower color. Blue flowers of *Lysimachia arvensis* perform better in terms of seedling mass or reproductive age in dry environments compared to a red morph (Arista et al., 2013). Schemske and Bierzychudek (2001) found a similar effect in *Linanthus parryae*, in that blue morphs produced more seeds than white morphs in years of low spring precipitation, possibly linked to bee preference. Since important pollinators like bees prefer blue hues, this suggests that in more stressful environments, selection might favor blue floral colors if possible, to provide resilience in the face of resource restrictions as to help attract presumably scarcer bee pollinators, as pollinator limitation is a major factor in plant reproductive success (Burd, 1994; Bennett et al., 2020; Giejsztowt et al., 2020). However, few studies have considered pollinator perception in such analyses, and below we attempt to synthesize available evidence to bridge the potentially contributing abiotic and biotic factors that might influence flower coloration. In this regard, recent work evaluating potential abiotic or biotic factors for the Australian continent reveals that both may be important, but biotic factors modeled with appropriate bee pollinator color space appear to be the main evolutionary driver of flower coloration (Dalrymple et al., 2020).

## Vision of Blue Across Species

**FIGURE 7:**
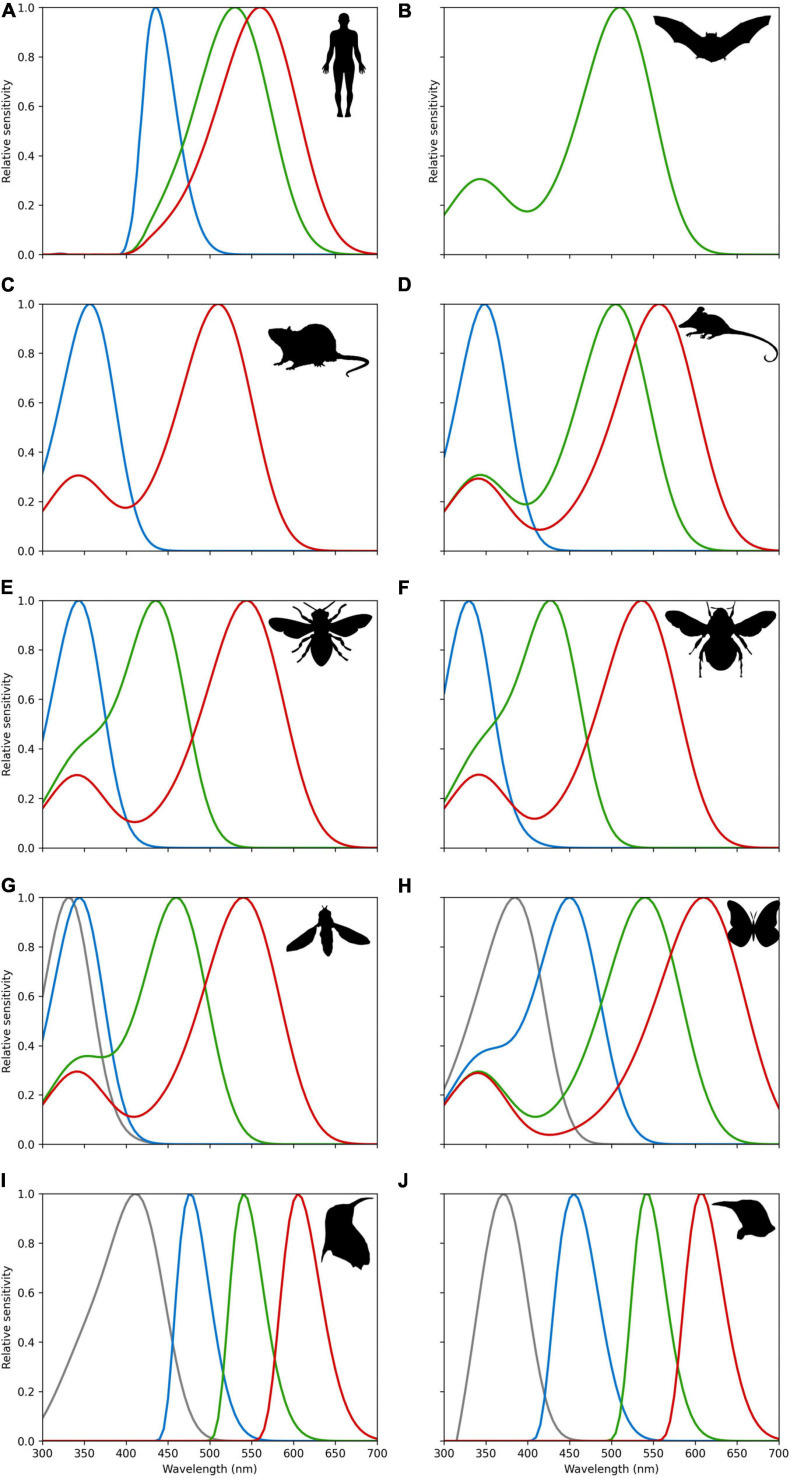
Spectral sensitivities of photoreceptors for **(A)** Trichromatic humans (*Homo sapiens*) (Dartnall et al., 1983; Stockman and Sharpe, 2000) as a point of comparison, and various animals know to visit flowers; **(B)** monochromatic flower bat (*Glossophaga soricine*) that senses short wavelength light via a secondary beta-band peak (Winter et al., 2003); **(C)** dichromatic mouse (*Mus musculus*) (Sun et al., 1997; Wester et al., 2009); **(D)** trichromatic Australian honey possum (*Tarsipes rostratus*) (Arrese et al., 2002); **(E)** trichromatic honeybee (*Apis mellifera*) (Peitsch et al., 1992; Briscoe and Chittka, 2001); **(F)** trichromatic bumble bee (*Bombus terrestris*) (Peitsch et al., 1992; Briscoe and Chittka, 2001); **(G)** tetrachromatic drone fly (*Eristalis tenax*) (Lunau, 2014); **(H)** tetrachromatic swallow tail butterfly (*Papilio aegeus*) (Matić, 1983); **(I)** tetrachromatic violet sensitive bird (Endler and Mielke, 2005; Hart and Hunt, 2007); **(J)** tetrachromatic ultraviolet sensitive bird (Endler and Mielke, 2005; Hart and Hunt, 2007). All sensitivities were modeled by implementing the Stavenga et al.’s (1993) vitamin A1 visual template namogram to enable easy comparison. It is known in humans and avians that ocular filtering modifies light reaching photoreceptors (Douglas and Marshall, 1999). For bird data, we thus also implemented generic ocular filtering functions representing typical ultraviolet and violet sensitive birds as proposed by Endler and Mielke (2005). For humans’ cornea and lens transmittance for a close relative primate species for which data are available (*Macaca fascicularis*). *Macaca fascicularis* data were extracted from data reported by Douglas and Marshall (1999). Line colors represent approximate region of the electromagnetic spectrum as typically perceived by humans. Relative sensitivity values for all species are provided in an electronic tabular form as Supplementary Material to enable future comparative research. Fly vision spectral sensitivity is mediated by green-sensitive photoreceptors coupled with UV-sensitive antennal pigments; thus, the spectral sensitivity of the four classes of R7/8 photoreceptors is likely to be relatively narrower and partially overlapping than the prediction here presented from the vitamin A1 template (Lunau, 2014). Detailed sensitivity data are available as Supplementary Appendix 2.

Human eyes are sensitive to light which lies in a very small region of the electromagnetic spectrum labeled “visible light.” This “visible light” corresponds to radiation with a wavelength range of about 400–700 nanometers (nm) which we perceive as a range of colors from violet through red. Three types of wavelength selective photoreceptors are responsible for our normal color vision. The blue (or S for Short) photoreceptor with a peak sensitivity at 421 nm, green (or M for Medium) photoreceptor at 530 nm, and red (or L for Long) photoreceptors at 559 nm (Stockman and Sharpe, 2000). Notable in the context of the discussion above (Figures 5, 6), humans do not actually have dedicated color photoreceptors for colors perceived as yellow. Our color perception is enabled by opponent color processing where red and green cone photoreceptor responses are processed in an opponent fashion with responses from our blue cone photoreceptor to generate the sensation of yellow, which is why we most typically see yellow and blue as opposite in representations like a color wheel (Hurvich, 1981). In a similar way to the blue preference in many adults discussed above, color perception is influenced by environmental effects (Palmer et al., 2013). This partially explains why color screening tests are done for driving a car, as, due to the complexity of human color vision, some individuals cannot tell the color difference between green and red, but still can discriminate some other colors (Backhaus et al., 1998; Stockman and Sharpe, 2000). Once we appreciate this point, it becomes questionable to what extent human color vision is appropriate for assessing flower colors that evolved for different animals, and a purpose of this manuscript is to provide a bridge between different ways of interpreting spectral data from flowers. Figure 7 shows, for example, the comparative visual system of different animals known to interact with flowering plants in a biologically relevant way, showing that what is “blue” is likely to be a perceptual dimension of a particular observer in many different ways. Some insect pollinators like butterflies and moths show evidence of a high level of diversity (Chen et al., 2013), even between genders of a single species (Arikawa et al., 2005). This variability is partly due to the complex genetics of the group where ommatidial types can be stochastically distributed in their eyes as shown for species belonging to two separate groups (Perry et al., 2016). For example, the trichromatic hawkmoth (*Manduca sexta*) shows a preference for blue (Goyret et al., 2008), other lepidopteran species like *Papilio xuthus* have six photoreceptor classes implicated in color vision that are also potentially subject to other light tuning factors that enable very difference color capabilities (Arikawa et al., 1999; Arikawa, 2003). Taken together with available evidence for some other important flower visitors (Figure 7), this shows that great care is required when considering how a flower color may appear to its biologically relevant pollinator.

## Plant–Pollinator Interactions

The main adaptive advantage promoting the evolution of specific flower colors is to capture the attention of preferred pollinators (Chittka and Menzel, 1992; Bukovac et al., 2017a), while also avoiding attention from other flower visitors that might only seek to rob flower rewards (Lunau et al., 2011; Camargo et al., 2019; Dyer and Shrestha, 2019). Darwin (1877) postulated that innate preferences might allow flower visitors to more easily find rewarding flowers, and indeed different insect clades have different innate color preferences, which likely represent phylogenetic adaptations of emerging insects foraging to first find rewarding flowers (Giurfa et al., 1995; Lunau and Maier, 1995; Raine and Chittka, 2005; Raine et al., 2006; Dyer et al., 2007, 2016a, 2019; Raine and Chittka, 2007; Ings et al., 2009; Morawetz et al., 2013; van der Kooi et al., 2019). Bees, for instance, have phylogenetically conserved trichromatic color vision with UV-, blue-, and green-receptors (e.g., Figures 7E,F), centering around 350, 440, and 540 nm, respectively (Chittka, 1996; Briscoe and Chittka, 2001; Dyer et al., 2012). Where two spectrally different photoreceptors overlap (e.g., around 400 nm in the blue end of the spectrum for bees), color resolution and learning is the strongest (von Helversen, 1972; Peitsch et al., 1992) and could promote color preferences (Menzel et al., 1974). For example, the common blue flowers in the order *Delphinium* are preferred by bees compared to relatively rarer white flower morphs, and the blue color appears to allow bees to see the flower better and thus results in the collection of more nectar rewards per unit time (Waser and Price, 1983). Indeed, experiments that artificially manipulate the color of such flowers with blue paint observe an increase in the efficiency with which both bumblebees and birds visit flower morphs (Waser and Price, 1985). In a similar way, wild-type flowers of *Antirrhinum majus* appear blue to bumblebees, and are both preferred and processed faster than genetically modified mutant flowers of bee-white appearance (Dyer et al., 2007). Nevertheless, it has also to be considered that many flowers are multi-colored, and the effects of such color patterning on pollinators and their preferences for different parts of the spectrum are complex and only starting to be explored (Lunau et al., 2016). In Germany, it was reported that blue flowers more frequently presented higher reward (Giurfa et al., 1995) than alternative flower colors. However, recent work in Australia reports no evidence that any particular color among bee-pollinated flowers was associated with higher nectar rewards (Shrestha et al., 2020), and so currently there is no conclusive evidence of higher rewards being associated with blue flowers.

According to Rodríguez-Gironés and Santamaría (2004), interactions among pollinators of different types might interfere with the expression of a color preference. When both bees and birds are in the same environment with equally rewarding blue or red flowers, birds may elect to preferentially visit red flowers since bees are taking rewards from the bee-preferred blue morphs, even though birds can efficiently process either color with their visual system. Thus, the difference in color visual systems among pollinators (see Figure 7) can influence which plant species may be successful in different environments, and such effects can be dynamic and depend upon the variety of flower visitors in an environment (Shrestha et al., 2013; Camargo et al., 2019), and how distributions may change due to factors like climate (Hegland et al., 2009; Shrestha et al., 2018). For example, when flower-visiting flies are the only pollinators in an environment like Macquarie Island in the Southern Ocean, all flowers from a wide range of plant families have been observed to reflect colors rich in long wavelength that are preferred by flies (Lunau, 2014; Shrestha et al., 2016). This strong effect consistent with pollination syndromes was also observed in a community in South Eastern Australia where orchids that were pollinated by bees more frequently had short wavelength blue colors, while fly-pollinated flowers in the same environment were never blue (Shrestha et al., 2019a).

## In Search of Blue: *Spectral Analysis and Bee Color Space*

**FIGURE 8:**
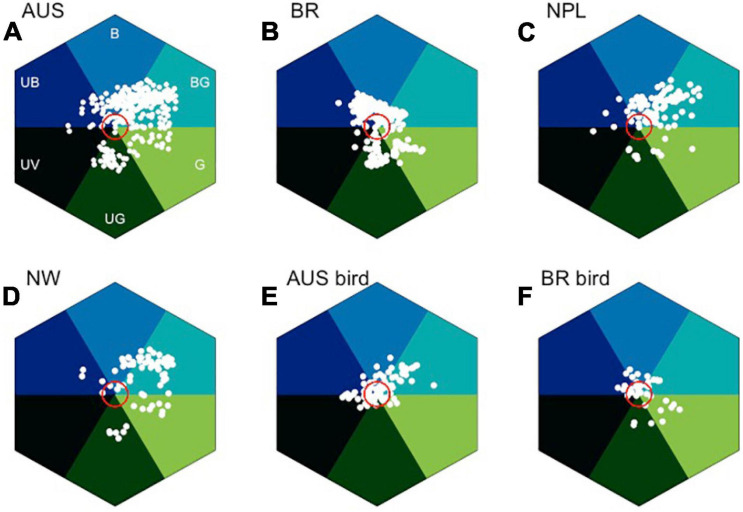
In search of blue: bee color space for four different countries, and also considering separately bird pollinated where quality data are available. **(A)** Australian insect-pollinated flowers (AUS, *n* = 146), **(B)** Brazilian bee-pollinated flowers (BR, *n* = 245), **(C)** Nepal (NPL, *n* = 107), **(D)** Norway (NW, *n* = 77), **(E)** Australian bird-pollinated flowers (*n* = 92), and **(F)** Brazilian hummingbird-pollinated flowers (*n* = 39). Red circle is 0.11 hexagon unit which shows the achromatic region where bees are predicted to be poor at detecting colors against a foliage background, showing that bird-pollinated flowers are more frequently achromatic considering bee color perception. Hex sector values are available in Supplementary Appendix 3.

To explore the importance of blue flower color and considering bee pollinator color vision, it is interesting to compare reflectance spectra of plants from different latitudes and geographic regions. Datasets from Australia (Dyer et al., 2012; Shrestha et al., 2019a,b), Nepal (Shrestha et al., 2014), Norway (Arnold et al., 2009, 2010), and Brazil (Camargo et al., 2019) provide an accessible comparison, because similar data collection methods were employed in the various studies, although some abiotic factors including background and/or light can vary with increasing altitude (Niu et al., 2020). The floral spectra from these studies were expressed in a bee color space, a geometrical interpretation allowing for modeling colors as perceived by an animal observer (Figure 8) (Kelber et al., 2003; Renoult et al., 2017); this model is implemented using a visual namogram (Stavenga et al., 1993) to model spectral sensitivity functions (Figure 7) for typical bee photoreceptors (350, 440, and 540 nm), using standard foliage background and open midday illumination (Judd et al., 1964), enabling the calculation of a spectral locus for the main flower color for each plant species (Figure 8).

In the bee color space proposed by Chittka (1992), flower loci for the Australian (Figure 8A), Brazilian (Figure 8B), Nepalese (Figure 8C), and Norwegian (Figure 8D) species are rare or non-existent in the “UV” category, but all other categories of the color hexagon do contain a significantly higher number of floral color signals. In the Australian data, where it has been possible to identify insect- or bird-pollinated flowers (Shrestha et al., 2013), very few insect-pollinated flowers have loci near the achromatic center of the bee color space that represents leaf foliage (Figure 8A). In contrast, many bird-pollinated flowers appear achromatic to bees (Figure 8D), and thus are difficult to be discriminated from the background and harder to detect by a bee observer. This difference results from the frequent evolution of red floral signals among bird-pollinated flowers (Shrestha et al., 2013; Burd et al., 2014), which tend to have spectral patterns that only weakly modulate bee photoreceptors (Lunau et al., 2011). It has been argued that the highly saturated red of bird-pollinated flowers has evolved to make flowers less apparent to bees and thus serving as bee avoidance mechanism (Lunau et al., 2011; Camargo et al., 2019; Coimbra et al., 2020). However, the spectral evidence could also be compatible with direct selection by birds, with the effect on bee perception arising as a by-product. For example, in Figure 8E, we see that in addition to the approximately 30% of flowers within the achromatic region of color space, the majority of bird-pollinated flowers can be visually detected by bees. Thus, more research on flower coloration using direct measurement of selection in field settings is required to dissect complex competing hypotheses of bee avoidance vs. selective pressure to evolve colors birds optimally process.

Color is a perception resulting from the particular way in which a brain processes visual information; therefore, it is important to use a model enabling for the interpretation of opponent processing to fully understand how color signaling is perceived (Chittka, 1992). Of the six sectors of bee color space, the most frequent flower color signals from Australia, Nepal, and Norway are in the “Blue-Green” (BG) category of color space (Figure 8), which is known to have high frequency of bee pollinated flowers (Chittka et al., 1994). In the Brazilian environment, the “Blue” (B) sector contains the most insect-pollinated flowers, followed by “Blue-Green” flowers (Figure 8B). A key reason for this short-wavelength preference is likely to be that by choosing blue colors, bees are able to reduce the effect of noisy signals resulting from long wavelength reflecting surfaces that are commonly found in nature (Bukovac et al., 2017b). The outstanding question then is why flowers categorized as being blue (B) as compared to blue-green (BG) are less frequent in the studied environments when considering biologically relevant bee observers (Figure 8)? A plausible explanation is that for pollinators to select a preferred color, they must first detect the color, which is a complex visual problem in natural environments (Bukovac et al., 2017a,b), a task which in bees is predominantly modulated by the achromatic processing channel of the long wavelength sensitive green photoreceptor (Giurfa et al., 1996; Dyer et al., 2008, 2016a,b; Wertlen et al., 2008; Skorupski and Chittka, 2011). Thus, flowers that from a bee’s perspective conform to these twin visual requirements need to modulate both blue and green photoreceptors. However, such colors should not modulate the UV receptor, as that would result in an achromatic stimulus that is difficult to discriminate from the background (Kevan et al., 1996, 2001; Waser and Chittka, 1998; Spaethe et al., 2001), which has been shown to be the case with gene-modified flowers (Dyer et al., 2007). This effect may appear counterintuitive to a human observer as we process brightness or intensity cues as a dimension of our color perception, but such a capacity is due to specialized neural circuitry in the primate visual system. In fact, evidence from honeybee studies shows that these pollinators do not reliably process brightness cues (Ng et al., 2018). Therefore, flower color loci lying in the “Blue-Green” (BG) sector of bee color space have spectra that bees both easily detect and innately prefer. This BG sector of bee color space represents the loci of human white flowers, which are also observed by our eye to be most frequent in nature (Figure 5). Many Australian and Brazilian bird-pollinated flowers also exist in this bee blue-green (BG) color sector (Figure 8E), but the flowers tend to be clustered toward the center of color space (Figure 8F). In addition to the importance of bee pollination, abiotic conditions may also be responsible for the predominance of “Blue” (B) flowers in the Brazilian environment (Figure 8B). In fact, the studied flower community corresponds to highly diverse mountain vegetation subjected to seasonally dry climate, high irradiance, and acidic soils with low nutrients content and high aluminum saturation (Silveira et al., 2016).

## Elevational Gradients

**FIGURE 9:**
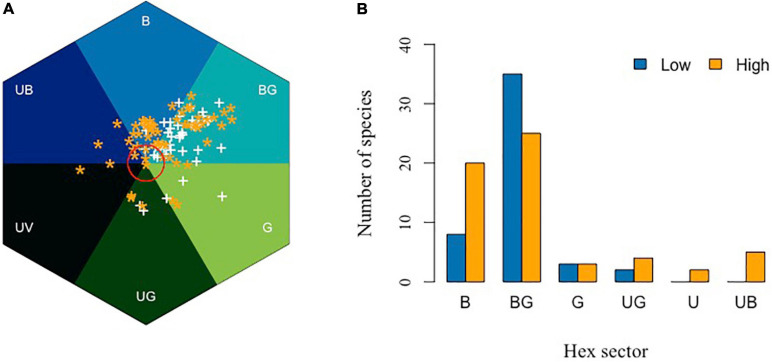
In search of blue in different elevation. **(A)** Bee color space for low (900–2000 m, white symbol “+”) and high (2700–4100 m, orange asterisk “*”) altitude of Nepal. Red circle is 0.11 hexagon unit which shows the achromatic region where bees are predicted to be poor at detecting colors against a foliage background. **(B)** Number of Himalayan plant species classified in the various hexagon categories of bee color space. Flower colors are not equally distributed across color sectors (Chi-square = 6.77, df = 1, *P* = 0.009), with a higher number of blue flowers being observed at higher altitudes (*z* = 2.76, *p* < 0.05). Data are available in Supplementary Appendix 3.

Flowers in higher elevations may also need to have more efficient color signaling to maximize the chance of attracting pollinators. Bees are efficient pollinators but do, in general, tend to be more frequent at lower elevations, while Diptera and Lepidoptera are more frequently observed at higher elevations (Arnold et al., 2009). Arnold et al. (2009) hypothesized that flower colors should shift from being more frequent in the B and UB categories of bee color space at low elevations, toward colors that reflect longer wavelengths at higher altitude due to a change in pollinator distributions, although their subsequent analyses of data up to 1600 m a.s.l. revealed no significant difference in flower coloration along an altitudinal gradient in Norway. In New Zealand, a high proportion of flowers in high altitudes displays colors perceived as being white by a human observer. However, recent research shows that while many mountain flowers in New Zealand are indeed white to a human observer, such flowers actually absorb UV and are thus highly chromatic for bees, lying in the BG category of bee color space, which is consistent with a preference for blue colors and reliable signal detections by bee pollinators (Bischoff et al., 2013). In the Himalayan mountains, it has been shown that floral colors were significantly more diverse at a high elevation (3000–4100 m a.s.l.) subalpine zone than in the subtropical zone (900–2000 m a.s.l.) of Nepal (Shrestha et al., 2014). Figure 9A shows the relative distribution of the flower colors from Nepal plotted considering the two altitudinal ranges, and shows that there is a significant shift toward shorter wavelength blue colors toward higher altitudes (Figure 9B).

**FIGURE 10:**
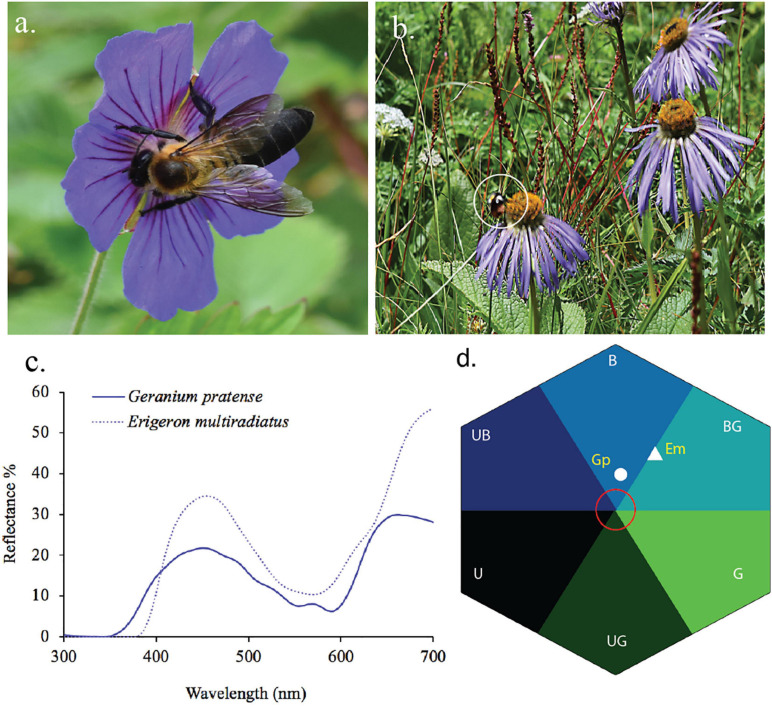
Blue flower and its pollinators from Nepalese Himalaya. **(a)** *Geranium pratense* (GP) visited by Himalayan native bees *Apis laboriosa*, **(b)** *Eriogeron multiradiatus* (Em) visited by native bumble bees (*Bombus* sp. shown in white circle), **(c)** reflectance spectra of the two aforementioned species, and **(d)** reflectance spectra converted into bee hexagon color space. In Nepal Himalaya, native bees are present up to 4200 m a.s.l. (Thapa, 2000) and bumblebees are available/found up to 5000 m a.s.l. including some part of Sikkim Himalaya (Williams et al., 2010; Shrestha et al., 2014; Streinzer et al., 2019).

Previous authors have observed and reported, based on human color vision, that with increasing altitude there appeared to be more blue flowers (Weevers, 1952), and by using modern pollinator observer models, it is possible to quantify evidence for such an effect (Figure 9). This observation leads to two lines of inquiry for the future to understand the significance of blue flower frequency with increasing altitude, including (i) are such observations consistent with the evidence from lower altitude reports that blue flowers are more frequent in harsher (e.g., drier; Schemske and Bierzychudek, 2001; Arista et al., 2013) environments and/or (ii) is it the presence of specialist high altitude bee pollinators (Figure 10) that promotes blue flower coloration? These hypotheses may not necessarily be mutually exclusive, as harsher conditions for plants likely also mean harsher conditions for insect pollinators. This, in turn, may create competition for pollinators, as plants are often pollinator limited in harsher conditions, potentially leading to a need for flowering plants to optimally advertise with the colors preferred by bees. Much work is required to understand what factors might lead to changes in flower color in different environments, and hopefully this review on fragmentary blue serves as a useful tool for bridging our understanding between how plants science and botany has classically thought of blue flower colors, and what the perception of such colors likely means for biologically relevant pollinators like bees.

## Conclusion

Our human color vision (Figure 7) enables seeing a small fragment of the electromagnetic spectrum. Through history humans have been attracted by the blue color which has been used to decorate items of economic or ritual value and inspired poets and artists. Our synthesis review shows that human blue flowers color is a rare color in nature (Figure 2A and Supplementary Figure 1), as reported by previous authors (Gottsberger and Gottlieb, 1981; Lee, 2010), and that the pathways enabling for the production of a blue coloration suggest that plant flower colors are potentially important biomarkers of changing environmental conditions like nutrient availability. However, human color vision is not an objective tool for the evaluation of color as it is perception highly variable between different individuals and context; for example, the famous blue/gold dress dilemma recently received widespread international attention and shows that human color vision sees the same stimuli as very different colors depending upon context (Winkler et al., 2015). Flowers did not evolve under the pressure of human color vision, so modeling pollinator vision with established techniques may provide a less biased insight into color mediated interaction between animal observers and plants that enable biotic pollination. Indeed, when considering harsh environments like high altitude in the Himalayan mountains, we observe that short wavelength blue flowers do indeed become more frequent (Figure 9), suggesting that biotic pollination is a key factor that must always be considered in mapping flower biodiversity. Thus, whenever considering biological factors influencing flowers color signaling, or including abiotic factors reported on by researchers, care must be taken as to what observer is most relevant to a particular question, and how different observers may need to be considered to understand how and why blue flower colors exist in complex natural environments.

## Author Contributions

All authors contributed equally based on their expertise in each section of this review manuscript.

## Conflict of Interest

The authors declare that the research was conducted in the absence of any commercial or financial relationships that could be construed as a potential conflict of interest.

## Supplementary Material

Supplementary Figure 1 Global flower color frequency across species based on the human visual system

Supplementary Appendix 1 R code for Figure 1.

Supplementary Appendix 2 Sensitivity data.

Supplementary Appendix 3 Hexagon sector data and R code.
