# Supplementary material for: Fragmentary Blue: Resolving the Rarity Paradox in Flower Colors
Source: Front Plant Sci. 2021 Jan 15;11:618203. doi: 10.3389/fpls.2020.618203 (PMC7859648; doi:10.3389/fpls.2020.618203)
Supplement: Supplementary Appendix 3 — Hexagon sector data and R code. [file Data_Sheet_4.ZIP › Supplementary Fig S1.docx]

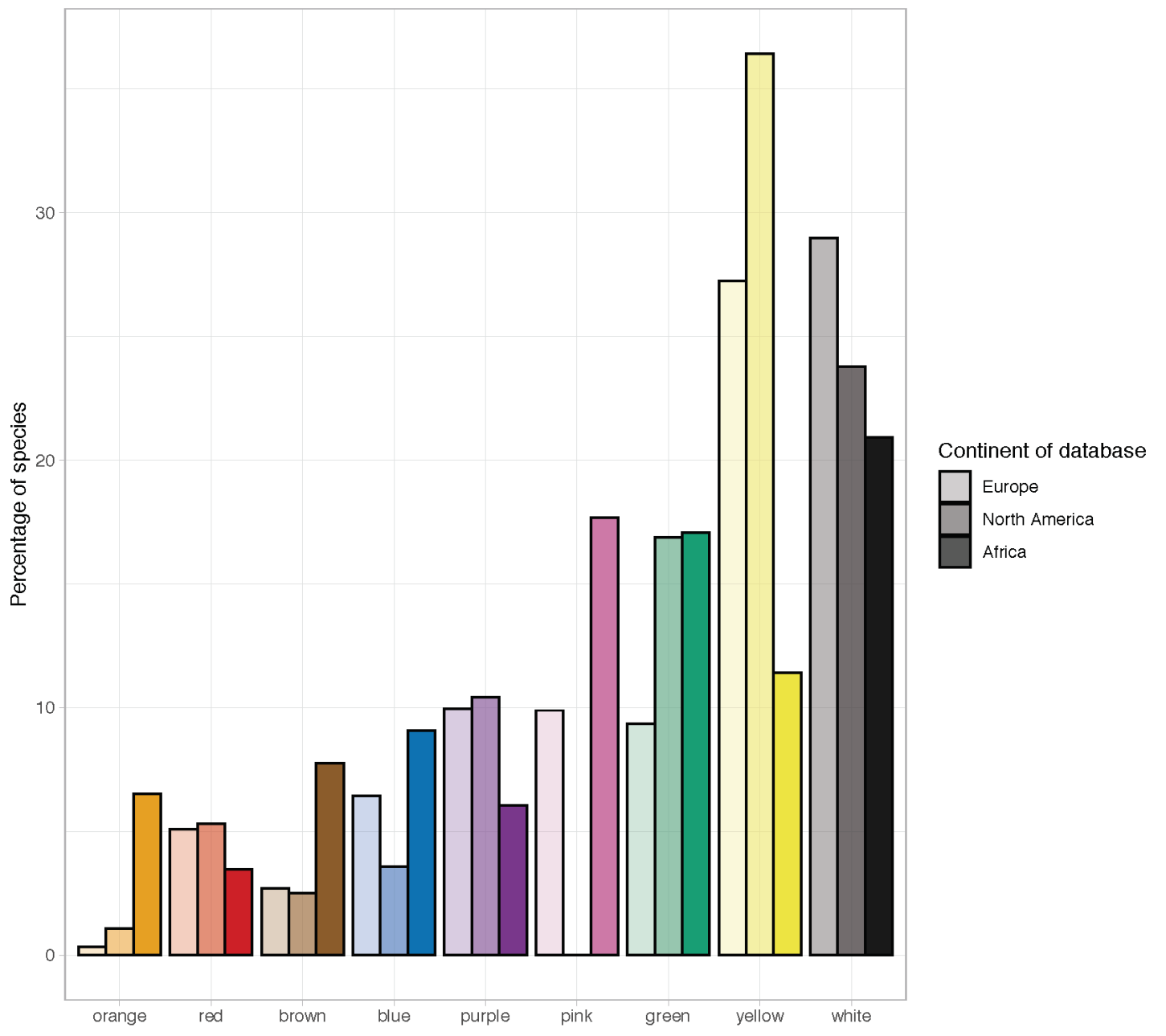


Supplementary **Fig S1**: Global flower color frequency across species based on the human visual system, shown separately for European (*n* =2852)., North American (*n* = 1842)., and African (*n* = 5743) datasets.  (Data source: Kattge et al., 2020, <https://www.try-db.org/TryWeb/)>.
